# Gas Expansion Three Days after Pars Plana Vitrectomy with Sulfur Hexafluoride 20% Tamponade following Carbon Monoxide Toxicity and Oxygen Therapy

**DOI:** 10.1155/2022/5035361

**Published:** 2022-05-09

**Authors:** Mojtaba Abrishami, Mehrdad Motamed Shariati, Ali Bolouki, Ghodsieh Zamani

**Affiliations:** Eye Research Center, Mashhad University of Medical Sciences, Mashhad, Iran

## Abstract

**Purpose:**

To report an unusual case of gas expansion following oxygen therapy in a patient with sulfur hexafluoride (SF_6_) 20% tamponade after pars plana vitrectomy. *Case Report*. A 40-year-old man came to the clinic with severe ocular pain and redness and also vision decrease in his left eye three days after uncomplicated 23-gauge pars plana vitrectomy, internal limiting membrane peeling combined with phacoemulsification, and using 20% SF_6_ injection as a tamponade agent due to significant cataract and a full-thickness macular hole. In ophthalmic examinations of his left eye, high intraocular pressure (approximately 70 mmHg), a flat anterior chamber, and a gas-filled vitreous cavity were found. The patient had been hospitalized the day before due to carbon monoxide poisoning and had undergone oxygen therapy with a pure 100% mask for three hours.

**Conclusion:**

It seems that oxygen therapy or carbon monoxide poisoning increases the volume of gas in the patient's vitreous cavity and the nonexpansile percentage of SF_6_ expands.

## 1. Introduction

Pars plana vitrectomy normally ends with an empty vitreous cavity which usually needs tamponade. The physical properties of the gases (air, sulfur hexafluoride (SF_6_), perfluoroethane (C_2_F_6_), and perfluoropropane (C_3_F_8_)) include interfacial surface tension and buoyancy, which have led to their use as tamponing agents in vitreoretinal surgeries for many years [1]. Because of the lower density, gas bubbles float on the surface of liquids. A small bubble of gas can cover a large area of the retina following the surgery [2]. The use of gases in vitreoretinal surgeries has known side effects such as raised intraocular pressure (IOP), gas migration, and cataract formation [3, 4]. Increased IOP is a vision-threatening complication following gas injection which mainly occurs due to gas expansion. The lower water solubility of pure gases (SF_6_, C_2_F_6_, and C_3_F_8_) than nitrogen leads to dissolving nitrogen in gas and increasing its volume. This is especially exacerbated when the patient is anesthetized with nitrous oxide (N_2_O) gas [2]. To prevent gas expansion, the surgeon dilutes the gas with air before injection [5]. The highest concentration of gas in the vitreous space that does not expand is called the isovolumic concentration. Studies have shown that this concentration is 20-25%, 16%, and 14-18% for SF_6_, C_2_F_6_, and C_3_F_8_, respectively [3, 5].

In this report, we are going to introduce an unusual case of gas expansion following oxygen therapy for carbon monoxide (CO) poisoning three days after pars plana vitrectomy with a nonexpansile percentage of diluted SF_6_ (20%) injection as a tamponade.

## 2. Case Presentation

**Figure 1:**
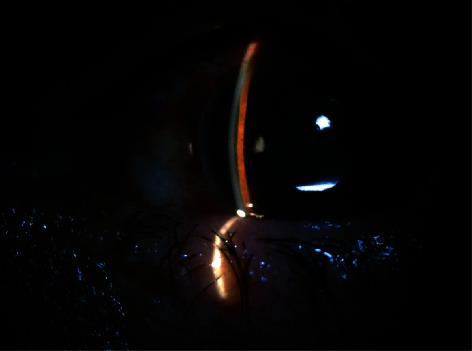
Forward dislocation of posterior chamber intraocular lens and iris with shallow anterior chamber.

A 40-year-old man came to the clinic because of decreased vision in his left eye. He underwent phacoemulsification with posterior chamber intraocular lens combined with 23-gauge pars plana vitrectomy, internal limiting membrane peeling, and 20% SF_6_ injection as a tamponading agent due to significant cataract and a full-thickness macular hole. The surgery was done under general anesthesia, and it was uncomplicated. The N_2_O gas was not used during the operation. After the fluid-air exchange, the surgeon used the following technique for gas-air exchange to ensure the accuracy of the injected gas concentration. Ten milliliters (ml) of pure SF_6_ (Alcon, Inc., Fort Worth, TX) was drawn up in a 50 ml syringe and diluted with room air through a Millipore filter. One of the sclerotomies was sutured with coated Vicryl (polyglactin 910) 7-0 suture (Ethicon, Johnson & Johnson, Raritan, NJ), and then, the 50 ml isovolumic concentration of SF_6_ (20% SF_6_) was flushed into the vitreous cavity through the other two sclerotomies. Finally, all sclerotomies were sutured while the intraocular pressure (IOP) seems normal. The morning after surgery, the patient was examined. Besides mild corneal edema, the rest of the examinations were unremarkable. The anterior chamber was deep and formed, and the vitreous cavity was filled with gas. The IOP was 12 mmHg. After 3 days, he referred with pain and redness in his left eye. The best-corrected visual acuity was hand motion (HM) detection. In ophthalmic examinations of his left eye, we found high IOP (approximately 70 mmHg with Goldmann applanation tonometer), diffuse corneal microcystic edema, and forward dislocation of the IOL and iris diaphragm with the flat anterior chamber, and a gas-filled vitreous cavity (Figure 1). The patient had been hospitalized the day before due to carbon monoxide poisoning and had undergone oxygen therapy with a pure 100% mask for three hours. We prepared the patient to be transferred to the operating room for gas adjustment surgery. Under sterile conditions and local anesthesia, 0.4 milliliters of gas was aspirated from the vitreous cavity with a 27-gauge needle in the operating room. Two hours after the surgery, the IOP reached 10 mmHg with no IOP lowering medications. In the next 2 weeks of follow-up examinations, there was no IOP rise and the patient needs no antihypertensive medications.

## 3. Discussion

Sulfur hexafluoride is a nontoxic, inert, and insoluble gas in aqueous humor. When pure SF_6_ is injected into the eye, it will expand because the rate at which nitrogen dissolves in gas is greater than the gas absorption into the surrounding tissue fluid compartment. This increase in volume stops when the concentration of nitrogen in the gas and blood reaches equilibrium. The absorption of the gas approximated a first-order kinetic equation concerning bubble volume [2].

During the expansion phase, intraocular pressure will rise and it will be sight threatening [6]. Using an isovolumic concentration of SF_6_ (20%) makes this complication the minimum. Even when the isovolumic concentration of gas is injected into the vitreous cavity, it is recommended not to use N_2_O for anesthesia [5]. In this case, due to not using N_2_O gas throughout the surgery, an increase in gas volume following the dissolution of nitrogen seems unlikely.

One of the most common post pars plana vitrectomy complications in gas-filled eyes is IOP rise [6–8]. Different mechanisms have been reported including open-angle due to gas expansion and angle-closure as a result of anterior displacement of the lens-iris diaphragm and iridocorneal apposition. Among the risk factors for increasing IOP are the patient's older age, the surgeon's mistake in estimating the gas concentration, and previous scleral buckle surgery [9–11]. This IOP increment can be extremely harmful to patients with impaired optic nerve head circulation. In our case, the surgeon used the most accurate method available to inject the gas at the desired concentration. As described earlier, in this method, first, the pure gas is diluted to the desired concentration and then lavaged inside the vitreous space. This technique is safer than injecting a small volume of pure gas into an air-filled eye to achieve a diluted gas mixture because the volume of the air in the vitreous cavity cannot be determined with sufficient accuracy. Furthermore, the patient's IOP was within the normal range on the first day after the surgery, while in the case of injection of higher concentrations of gas, we should have seen an increase in IOP during this period [11]. It can be concluded that the hypothesis of increasing the gas volume due to injection of incorrect concentration seems unlikely.

Although we do not know the exact amount and duration of the patient's treatment with oxygen, it seems that oxygen therapy following carbon monoxide poisoning increases the volume of gas in the patient's eye three days after the surgery. Oxygen (O_2_) therapy in patients with CO poisoning separates CO molecules from hemoglobin (Hb) by competition mechanism. This process occurs in the respiratory system. Besides, the binding of the CO molecules to Hb is much stronger than that of O_2_ to Hb [12]. According to this knowledge, the rate of release of CO molecules from Hb in peripheral tissues is low and the possibility of gas expansion due to the dissolution of CO molecules seems illogical. Following O_2_ therapy, a higher percentage of Hb is saturated with oxygen, and it is possible that an increase in gas volume occurred following the dissolution of the released oxygen in the vitreous cavity.

Given that increased IOP following gas dilation can be potentially blinding, it is best to warn patients and other clinical staff to consult with an ophthalmologist for a vitrectomized and gas-filled patient who needs oxygen therapy.

## Data Availability

The datasets used during the current study are available from the corresponding author on reasonable request.
